# Adult neurogenesis in the telencephalon of the lizard *Podarcis liolepis*

**DOI:** 10.3389/fnins.2023.1125999

**Published:** 2023-02-23

**Authors:** Susana González-Granero, Enrique Font, Ester Desfilis, Vicente Herranz-Pérez, José Manuel García-Verdugo

**Affiliations:** ^1^Laboratory of Comparative Neurobiology, Cavanilles Institute of Biodiversity and Evolutionary Biology, University of Valencia and CIBERNED-ISCIII, Valencia, Spain; ^2^Ethology Lab, Cavanilles Institute of Biodiversity and Evolutionary Biology, University of Valencia, Valencia, Spain; ^3^Laboratory of Evolutionary and Developmental Neurobiology, Department of Experimental Medicine, Lleida’s Institute for Biomedical Research-Dr. Pifarré Foundation (IRBLleida), University of Lleida, Lleida, Spain; ^4^Department of Cell Biology, Functional Biology and Physical Anthropology, University of Valencia, Burjassot, Spain

**Keywords:** adult neurogenesis, reptiles, lizard, neural stem cells, neuroblast migration, radial glia, sulci, tangential migration

## Abstract

In adult lizards, new neurons are generated from neural stem cells in the ventricular zone of the lateral ventricles. These new neurons migrate and integrate into the main telencephalic subdivisions. In this work we have studied adult neurogenesis in the lizard *Podarcis liolepis* (formerly *Podarcis hispanica*) by administering [^3^H]-thymidine and bromodeoxyuridine as proliferation markers and euthanizing the animals at different survival times to determine the identity of progenitor cells and to study their lineage derivatives. After short survival times, only type B cells are labeled, suggesting that they are neural stem cells. Three days after administration, some type A cells are labeled, corresponding to recently formed neuroblasts. Type A cells migrate to their final destinations, where they differentiate into mature neurons and integrate into functional circuits. Our results after long survival periods suggest that, in addition to actively dividing type B cells, there is also a type B subpopulation with low proliferative activity. We also found that new neurons incorporated into the olfactory bulb are generated both *in situ*, in the walls of the anterior extension of the lateral ventricle of the olfactory bulbs, but also at more caudal levels, most likely in anterior levels of the sulcus ventralis/terminalis. These cells follow a tangential migration toward the olfactory bulbs where they integrate. We hypothesized that at least part of the newly generated neurons would undergo a specialization process over time. In support of this prediction, we found two neuronal populations in the cellular layer of the medial cortex, which we named type I and II neurons. At intermediate survival times (1 month) only type II neurons were labeled with [^3^H]-thymidine, while at longer survival times (3, 6, or 12 months) both type I and type II neurons were labeled. This study sheds light on the ultrastructural characteristics of the ventricular zone of *P. liolepis* as a neurogenic niche, and adds to our knowledge of the processes whereby newly generated neurons in the adult brain migrate and integrate into their final destinations.

## 1. Introduction

**FIGURE 1:**
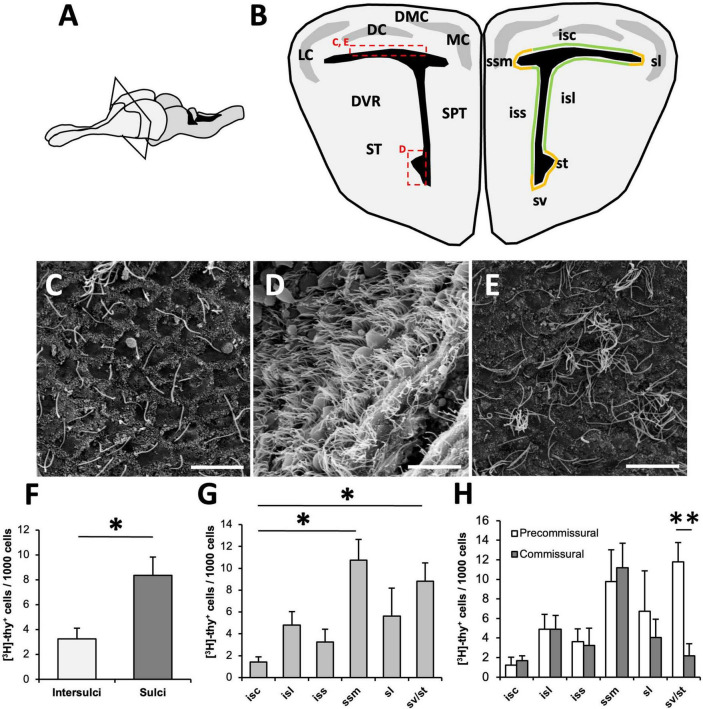
Organization of the ventricular zone (VZ) in *Podarcis liolepis*. **(A)** Schematic of the brain of *P. liolepis*. The telencephalon is represented in a lighter shade. **(B)** Diagram of a transverse section of the telencephalon at an intermediate pre-commissural level. In the left hemisphere are represented the main telencephalic regions, while in the right hemisphere shows the regionalization of the VZ in sulci (orange) and intersulcal regions (green), divided as sulcus septomedialis, sulcus lateralis, sulcus ventralis, and sulcus terminalis. Insets indicate the approximate location of the images shown in panels **(C–E)**. **(C)** Scanning electron microscopy (SEM) image showing the surface of the dorsal wall of the lateral ventricle (LV). In this region most cells are uniciliated. **(D)** SEM image of the surface of the LV at the level of the sv/st where most cells are multiciliated. **(E)** SEM image of the LV dorsal wall surface, in which some clusters of multiciliated cells intermingled with uniciliated cells can be observed. **(F)** Regional distribution of [^3^H]-thymidine-positive ([^3^H]-thy^+^) cells 1.5 h after [^3^H]-thymidine administration. Proliferative cells are more frequently found in sulci than in intersulcal regions (*n* = 5, two-tailed paired *t*-test). **(G)** Graph showing the distribution of [^3^H]-thy^+^ cells after 1.5 h of survival within the different sulci and intersulcal regions of the LV. The sulcus septomedialis and the sulci ventralis/terminalis presented a significantly higher number of proliferating cells than the intersulcus corticalis (*n* = 5, Friedman’s test followed by Dunn’s multiple comparisons test). **(H)** Distribution of [^3^H]-thy^+^ cells after 1.5 h of survival in the different sulci and intersulcal regions comparing pre-commissural and commissural levels. Proliferating cells were equally distributed in anterior and posterior levels in all regions except in the sulci ventralis/terminalis, with a higher concentration of [^3^H]-thy^+^ cells in anterior levels than in caudal levels (*n* = 5, two-tailed paired *t*-test). DC, dorsal cortex; DMC, dorsomedial cortex; DVR, dorsal ventricular ridge; isc, intersulcus corticalis; isl, intersulcus lateralis; iss, intersulcus septalis; LC, lateral cortex; MC, medial cortex; sl, sulcus lateralis; SPT, septum; ssm, sulcus septomedialis; ST, striatum; st, sulcus terminalis; sv, sulcus ventralis. Data are represented as the mean ± SEM. *p < 0.05, ^**^p < 0.01. Scale bars: 10 μm.

Adult neurogenesis is the process by which neurons are generated from neural stem cells in the adult brain, either to add or to replace neurons in pre-existing circuits. This phenomenon has been shown to take place in all vertebrate groups: fish (Birse et al., 1980; Zupanc and Horschke, 1995), amphibians (Easter, 1983; Polenov and Chetverukhin, 1993), reptiles (Lopez-Garcia et al., 1988; Holtzman and Halpern, 1991; Pérez-Canellas and García-Verdugo, 1992), birds (Nottebohm and Alvarez-Buylla, 1993) and mammals (Altman and Das, 1965; Kaplan and Hinds, 1977), including humans (Eriksson et al., 1998; Sanai et al., 2004). The telencephalon seems to be the main region in which this phenomenon occurs in all vertebrates, especially in amniotes. However, adult neurogenesis has been shown to affect different vertebrate groups to different extents. Based on the different species studied during the last decades, adult neurogenesis in non-mammalian vertebrates is much more extensive than in mammals in terms of the number of brain areas affected, as well as in the number of neurons produced. Thus, while in mammals adult neurogenesis is restricted to the olfactory bulbs (OB) (Bayer, 1983) and the hippocampal dentate gyrus (Altman and Das, 1965), in lizards this process affects, to a greater or lesser extent, the main telencephalic subdivisions (Pérez-Cañellas et al., 1997): the OB (main and accessory), cerebral cortex, dorsal ventricular ridge (DVR), striatum, septum, and nucleus sphericus (Figures 1A, B).

In most groups of amniotes, neural stem cells are located in the ventricular walls, mainly in those of the lateral ventricles (LV) (García-Verdugo et al., 2002), except for the mammalian hippocampus, in which stem cells are located in the subgranular portion of the dentate fascia (Seri et al., 2001). In some lizards (e.g., *Lacerta agilis*, *Eublepharis macularius*, and *Tarentola mauritanica*), proliferating cells seem to be mainly aggregated in discrete regions of the ventricular zone (VZ) of the telencephalic LV, known as *sulci* (Schulz, 1969; yanes-Méndez et al., 1988a). In these species, four sulci have been identified: septomedialis, lateralis, ventralis, and terminalis (Figure 1B). These sulci are thought to contain remnant cells of embryonic germ tissue that maintain their proliferative potential in the adult (Schulz, 1969; Tineo et al., 1987). Sulci are cytoarchitecturally characterized by the presence of a pseudostratified epithelium of columnar cells, unlike the rest of the VZ where a monostratified epithelium with cubic or flattened cells is found (Yanes-Méndez et al., 1988b), although the latter also presents some proliferative activity. Depending on the species studied, the relative abundance of proliferating cells in sulci compared to intersulcal regions is more or less pronounced (Font et al., 2001).

In the walls of the lateral ventricles of lizards three cell types have been identified: type B cells, type E cells and type A cells (García-Verdugo et al., 2002). Type B and type E cells are both radial glia cells, positive to GFAP, in contact with the ventricular lumen, and with very similar ultrastructural characteristics. However, their distribution on the ventricular surface is yet unknown. Both cell types are characterized by a radial process that is usually divided into two or more branches and appears to extend through the brain parenchyma to the pial surface. This contact with the pial surface is maintained throughout life and supports the radial migration of neuroblasts (Lazzari and Franceschini, 2001). The main difference between these two cell types is that B cells present a single primary cilium in the ventricular surface, whereas E cells are multiciliated. In contrast, type A cells are not in direct contact with the ventricle. These cells present relaxed chromatin in their nucleus, few cytoplasmic organelles and abundant polyribosomes and microtubules. The ultrastructural characteristics of type A cells are the same as those of the migrating cells found in the inner plexiform layer of the medial cortex (MC) (Garcia-Verdugo et al., 1986). Previous studies using proliferation markers in lizards have noted that at short survival times dividing cells always seem to be in contact with the ventricle. Therefore, the available evidence suggests that radial glia, or a subpopulation of this cell type, act as primary neural precursors in these species (Font et al., 1995; Pérez-Cañellas and García-Verdugo, 1996).

In lizards, proliferation in the VZ generates essentially neurons but not glial cells (Lopez-Garcia et al., 1988; Pérez-Cañellas and García-Verdugo, 1996). This is supported by the fact that, under normal conditions, free glial cells are very scarce in the telencephalon (Font et al., 2001), with astrocyte functions being performed by the radial glia that line the ventricles (García Verdugo et al., 1981). This differs from the situation in turtles, in which both cell types are produced (Pérez-Cañellas et al., 1997).

Newly generated neurons migrate through the brain parenchyma to their final destination, where they differentiate into neurons and integrate into pre-existing neuronal circuits (Lopez-Garcia et al., 1990). In general, new neurons migrate radially for short distances since their final location is usually next to the proliferating VZ where they are generated. During their migration, they use radial glia processes as a scaffold (Garcia-Verdugo et al., 1986; Lopez-Garcia et al., 1988). However, there is an exception to this general migration process: the migration of new neurons toward the OB. According to different studies in several lizard species, it appears that most, if not all, new neurons incorporated into this region are not generated *in situ*, since with short survival times after administration of a proliferation marker, labeled cells are rarely detected in the ventricular walls of the OB (Peñafiel et al., 1996; Pérez-Cañellas and García-Verdugo, 1996; Pérez-Cañellas et al., 1997).

Because of its purported homology with the dentate gyrus of the mammalian hippocampus, one of the regions that has received more attention in the telencephalon of lizards is the medial cortex (Olucha et al., 1988). Interestingly, this region concentrates much of adult neurogenesis in lizards (Font et al., 2001; Marchioro et al., 2005). The organization of the MC, like the rest of cortical areas in lizards, is relatively simple, with three layers: the inner plexiform layer, limited ventrally by the VZ, the cell layer, where most of the neurons of the MC are located, and the outer plexiform layer, limited dorsally by the meninges (Ebbesson and Voneida, 1969). Based on their ultrastructural features, two types of neuronal populations have been identified in the cell layer of the MC: small neurons (type I) and large neurons (type II) (Garcia Verdugo et al., 1984; Davila et al., 1985). Type I neurons are characterized by a small, round nucleus with clumped chromatin and scant cytoplasm. Type II neurons, in contrast, have a large oval nucleus with relaxed chromatin and a prominent nucleolus, abundant organelle-rich cytoplasm with numerous polyribosomes, and microtubules. It has been shown that in the MC of adult lizards type I neurons predominate, while in juveniles there is a greater proportion of type II neurons (López-García et al., 1984). Cell proliferation studies have shown that after 1 month after administration of a proliferation marker, only type II cells are marked (Pérez-Cañellas and García-Verdugo, 1996; Font et al., 2001). Nevertheless, the origin of type I neurons is still unknown.

In this study, we conduct an ultrastructural characterization of the neural precursors present in the VZ of the lizard *Podarcis liolepis* (formerly known as *Podarcis hispanica*). By administering [^3^H]-thymidine and bromodeoxyuridine as proliferation markers and euthanizing the animals at different survival times, we sought to determine the identity of progenitor cells and their lineage derivatives, including neurons incorporated into the OB and in the cellular layer of the MC. The generation of new neurons around the OB extension of the LV has been reported to be scarce but not negligible (Peñafiel et al., 1996; Pérez-Cañellas and García-Verdugo, 1996), thereby making it difficult to estimate the potential incorporation of migrating new neurons generated in more caudal levels of the VZ. For this reason, we performed an experiment in which we surgically excised the OB from the rest of the telencephalon by sectioning at the level of the olfactory peduncle, preventing any cells from the anterior telencephalic levels from reaching the OB. Our results indicate that proliferative activity in the VZ corresponds to type B cells, and suggest that many of the new neurons incorporated into the OB originate caudally, most likely from anterior levels of the sulcus ventralis/terminalis, and reach the OB by tangential migration. Finally, we also conclude that there is a continuous incorporation of type II neurons that progressively mature giving rise to type I neurons in the MC of this species.

## 2. Materials and methods

### 2.1. Animals

The animals used in this work were captured, maintained, and treated in accordance with the Spanish legislation, with authorization for the use for research purposes from the Valencian Council of Territory and Habitat (registry number 2007/4900). Experimental animals were adult Catalonian wall lizards (45–60 mm snout-vent length), *P. liolepis* (Sauria, Lacertidae), wild-caught in the province of Valencia (Spain) between the years 2007 and 2010. In all experiments, we used both male and female lizards indistinctively unless otherwise stated.

Prior to, and during the experimentation, the lizards were maintained in terraria with free access to water and a 14 h light/10 h dark cycle. The lizards were fed mealworms (*Tenebrio molitor* larvae) dusted with a vitamin supplement (Multicentrum, GSK) three times a week and occasionally with aphids (*Acyrthosiphon pisum).*

### 2.2. Scanning electron microscopy

For these experiments we used untreated adult lizards (*n* = 5). Animals were deeply anesthetized with halothane (2-bromo-2-chloro-1,1,1-trifluoroethane, Sigma, San Luis, MO, USA) and decapitated. The brains were removed, and the VZ surrounding the LVs were dissected out, obtaining three regions: cortical, dorsal ventricular ridge/striatum (DVR/St), and septal. The resulting whole-mounts were fixed by immersion in 2.5% glutaraldehyde (GA) and 2% paraformaldehyde (PFA) for 1 hour (h), washed twice in 0.1 M phosphate buffer (PB), pH 7.4, and incubated for 1 h in 1% osmium tetroxide in 0.1 M PB. Tissues were dehydrated first through ethanol series and then with CO_2_ using the critical point drying method. The samples were coated with gold/palladium alloy by sputter coating and examined under a scanning electron microscope Hitachi (S-4100; Hitachi, Tokyo, Japan).

### 2.3. Treatment with [^3^H]-thymidine and autoradiography

Lizards received intraperitoneal injections of [^3^H]-thymidine (Amersham; specific activity 5 Ci/mmol). Depending on their survival time, the final dose was different. For survival times of 1.5 h to 3 days, the animals received a single injection with a dose of 5 μCi/g body weight (b. wt.), while for longer survival times (1–12 months) animals received daily injections (5 μCi/g b. wt.) during three consecutive days, up to a total dose of 15 μCi/g b. wt.

For short survival times, we injected four lizards (*n* = 4) for each survival time (6, 12, 24, and 72 h), except for the 1.5 h survival time, for which we injected five (*n* = 5). For long survival times (1, 3, 6, and 12 months) we injected 3 animals for each time (*n* = 3).

Following their corresponding survival time, the animals were deeply anesthetized with Ketolar (ketamine hydrochloride, 0.6 mg/g b. wt.) and perfused with saline (0.9% NaCl), followed by a fixative consisting of 4% PFA and 2% GA. The complete bodies of the lizards were postfixed in the same fixative during 24 h. The brains were removed from the skull, sectioned frontally or longitudinally on a vibratome at 200 μm, postfixed in 2% osmium tetroxide for 2 h, rinsed, dehydrated, and embedded in epoxy resin (Durcupan, Sigma, San Luis, MO, USA). Semithin sections were cut at 1.5 μm with an ultramicrotome (UC6 Ultracut, Leica, Wetzlar, Germany) and mounted on gelatin-coated glass-slides, which were dipped in LM-1 hypercoat emulsion (Amersham), dried in the dark, and stored at 4°C for 30 days. The autoradiographs were developed using standard methods and counterstained with 1% toluidine blue.

### 2.4. Transmission electron microscopy of [^3^H]-thymidine labeled cells

For each animal treated with [^3^H]-thymidine, a minimum of 45 semithin section were examined for the presence of [^3^H]-thymidine labeled cells and photographed under a Nikon microscope (Eclipse E800, Nikon with digital camera Nikon DS-Ri1). A cell was considered labeled if it had eight or more silver grains over the nucleus. The semithin sections containing the selected cells were re-embedded, and ultrathin sections were cut and examined under a FEI Tecnai G^2^ Spirit transmission electron microscope (FEI Company, Tokyo, Japan). Images were acquired using Radius software (Version 2.1) with a XAROSA digital camera (EMSIS GmbH, Münster, Germany).

### 2.5. Quantitative analysis of [^3^H]-thymidine labeled cells

To assess the distribution of proliferating cells in the VZ, lizards injected with [^3^H]-thymidine with a survival time of 1.5 h were used (*n* = 5). The VZ was divided in six different regions, including three sulcal zones (sulcus medalis, sulcus lateralis and sulcus ventralis/terminalis) and three intersulcal zones (intersulcus corticalis, intersulcus lateralis, and intersulcus septalis) (Figure 1B). The number of [^3^H]-thymidine labeled cells was counted in all these regions relative to the total number of cells. This quantification was performed in two telencephalic levels: one pre-commissural (anterior) and one post-commissural (posterior), analyzing for each level a total of 7 semithin sections which were 9 μm apart to avoid counting the same cell twice. Different types of counts were performed by quantifying the total number of labeled cells/1000 cells considering sulci vs. intersulcal regions, comparing between the different sulci and intersulcal regions and differentiating between the pre- and post-commissural levels for each animal.

To characterize the ultrastructure of VZ proliferative cells and their derivatives, the brains of specimens with 1.5, 6, 12, 24, and 72 h survival times were examined. Between 50 and 150 [^3^H]-thymidine-positive ([^3^H]-thy^+^) cells were analyzed for each survival time, including at least two different antero-posterior levels per lizard. These cells were studied by transmission electron microscopy (TEM) to determine their ultrastructural characteristics. Counts were also made of the number of cells in mitosis (M phase) labeled relative to the total number of [^3^H]-thy^+^ cells.

The analysis of specimens with long survival times (1, 3, 6, and 12 months) focused mainly on the cell layer of the MC, although we also investigated whether there were labeled cells in the walls of the LVs. Within the MC we analyzed the ultrastructure of 25–50 [^3^H]-thy^+^ cells from each survival time to see to which neuronal type they corresponded.

### 2.6. Treatment with bromodeoxyuridine

A group of lizards (*n* = 6) received a single intraperitoneal injection of 5-bromo-2′-deoxyuridine (BrdU; 100 mg/kg b. wt., Sigma, San Luis, MO, USA) and were euthanized after survival periods of 1.5 h (*n* = 3) and 3 days (*n* = 3). A second group of lizards (*n* = 3) received daily injections of BrdU (100 mg/kg b. wt.) during five consecutive days up to a total dose of 500 mg/kg and were euthanized 7 days after the first injection. After their corresponding survival period, the animals were deeply anesthetized with Ketolar (0.6 mg/g b. wt.) and perfused transcardially with saline followed by 4% PFA in 0.1 M PB, pH 7.4. Then the complete bodies were postfixed in the same fixative solution for 6 h. Subsequently, the brains were removed from the skull, dehydrated, and embedded in paraffin. Transverse or longitudinal sections of the brain were cut at 10 μm and mounted on gelatin-coated glass-slides.

### 2.7. Fluorescence immunohistochemistry

Two types of immunofluorescence detections were performed on the BrdU-treated lizards. For those in the first group, with survival times of 1.5 h or 3 days (*n* = 3 each), a triple immunofluorescence detection for GFAP/DCX/BrdU was performed. For those in the second group, with a survival time of 7 days from the first injection and 3 days from the last injection (*n* = 3), a double immunofluorescence for BrdU/PCNA was performed.

In both cases the protocol for fluorescence immunohistochemistry was similar, except for the antibodies used. First, the slides were deparaffinized and hydrated. Then, they were treated with HCl 2N for 10 min at 37°C for DNA denaturation, rinsed in 0.1 M borate buffer and washed in phosphate buffered saline containing 0.1% Triton X-100 and BSA 0.1% (PTA). Subsequently, the sections were incubated in a blocking solution containing 10% casein (Vector) or 5% normal goat serum (NGS) (Sigma, San Luis, MO, USA) in PTA for 1 h, for triple or double immunoassay, respectively. After rinsing in PTA, the sections were incubated in blocking solution with the corresponding primary antibodies overnight at 4°C. The primary antibodies used for the first group were: mouse anti-BrdU (1:150, Dako), rabbit anti-GFAP (1:500, Dako), and goat anti-DCX (1:200, Sta. Cruz Biotechnologies); and for the second group: mouse anti-PCNA (1:500, Sigma, San Luis, MO, USA), and rat anti-BrdU (1:200, Abcam, Cambridge, UK). Sections were then washed with PTA and incubated with fluorescent secondary antibodies at 1:500 in blocking solution for 1 h at room temperature in the dark. The secondary antibodies used for the first group were: donkey anti-mouse Alexa 647 (1:500, Invitrogen, Walthan, MA, USA), donkey anti-rabbit Alexa 488 (1:500, Invitrogen, Walthan, MA, USA), and donkey anti-goat Alexa 555 (1:500, Invitrogen, Walthan, MA, USA); and for the second group: goat anti-mouse Alexa 555 (1:500, Invitrogen, Walthan, MA, USA), and goat anti-rat Alexa 488 (1:500, Invitrogen, Walthan, MA, USA). The sections were then washed in 0.1 M PB and incubated for 10 min with DAPI 1:1000 in H_2_O (Sigma, San Luis, MO, USA) at room temperature in the dark. Finally, the slides were washed with 0.1 M PB and mounted with Fluorsave (Calbiochem). The sections were analyzed with a Leica (Wetzlar, Germany) SP2 TCS AOBS inverted confocal microscope.

### 2.8. Post-embedding immunocytochemistry

We studied the colocalization of [^3^H]-thymidine-labeled cells with DCX or GFAP in the experimental groups with survival times of 1.5 h and 3 days after administration of [^3^H]-thymidine. For this purpose, we obtained four series of semithin sections of three telencephalic levels: pre-commissural telencephalon, anterior olfactory nucleus (AON), and olfactory peduncle (sectioned longitudinally). In the first and third series we performed autoradiographic detections of [^3^H]-thymidine labeled cells. In the second and fourth series we performed immunohistochemistry detection for DCX and GFAP, respectively. Post-embedding immunohistochemistry was performed as previously described (Crespo et al., 2003). The primary antibodies used were polyclonal rabbit anti-glial fibrillary acidic protein (1:500, Dako) or polyclonal goat anti-doublecortin (1:200, Santa Cruz Biotechnology, Dallas, TX, USA). The secondary antibodies were biotinylated goat anti-rabbit and biotinylated rabbit anti-goat (Vector laboratories), respectively. We then performed studies correlating DCX- and GFAP-positive cells with the analysis of their ultrastructure in adjacent semithin sections in which they were co-labeled with [^3^H]-thymidine.

### 2.9. Surgical sectioning of the olfactory peduncle

For this experiment, a total of 10 adult lizards of both sexes were used. Five animals underwent a complete olfactory peduncle sectioning procedure (*n* = 5) while the rest of them (*n* = 5) were subjected to a sham surgery. In both cases the animals were anesthetized with ketamine hydrochloride (Ketolar) at a concentration of 375 μg/g b. wt. The two frontoparietal scales, located between the supraocular scales and the pineal eye, were lifted, the skull was pierced centrally, and the meninges were gently removed. In one of the groups, the olfactory peduncle was sectioned bilaterally and Gelfoam (Pfizer, New York, NY, USA) applied between the resulting ends, while in the other (control group) Gelfoam was placed on top of the olfactory peduncles without sectioning them. Then, the skull window was closed using bone wax.

Animals were maintained for 2 weeks after surgery to allow them to recover. Then they were injected with 5 μCi/g b. wt. of [^3^H]-thymidine for 3 consecutive days, receiving a total dose of 15 μCi/g b. wt. Lizards were allowed 1 month of survival after [^3^H]-thymidine administration.

One of the OBs from each specimen was processed for electron microscopy and embedded in epoxy resin as described above. From a randomly selected starting level, 30 semithin sections (1.5 μm thick) were obtained and analyzed, corresponding to 45 μm. Following this procedure, successive series obtained every 200 μm were studied to cover the whole OB. Autoradiographic detection was performed on these sections as described in the corresponding section.

### 2.10. Stereological analysis

For stereological quantification, initial estimations of the total volume of the olfactory bulbs, main olfactory bulb (MOB) and accessory olfactory bulb (AOB) were performed. To do so, we used the Cavalieri method by measuring the area of the first semithin section of each level (A) included in each region (MOB or AOB), considering that there was a separation of 200 μm (t_*ref*_) between levels, thus obtaining the reference volume (V_*ref*_).


$$V_{r\,e\,f}=t_{r\,e\,f}\cdot \sum_{i=1}^{n}A_{i}$$

The point for sectioning the first level was decided randomly. The number of levels (i) comprising each region for the OB, MOB, and AOB was different for each animal.

Next, [^3^H]-thy^+^cells were counted using the physical dissector. For this purpose, we took 4 pairs of semithin sections from each level separated by 12 μm, and the slices of each pair of semithin sections were separated by 3 μm (t_*dis*_). We counted the cells that were labeled in one slice and not in the next, first in one direction (Q_1_) and then in the opposite (Q_2_), analyzing a total of 8 dissectors per level (d), Q being the total number of cells counted for each animal. The dissector volume (V_*dis*_) was then obtained by adding the volumes of each analyzed level.


$$Q=Q_{1}+Q_{2}$$

$$V_{d\,i\,s}=t_{d\,i\,s}\cdot \sum_{i=1}^{n}A_{i}$$

Finally, the total number of [^3^H]-thy^+^ cells (N) for each region in the OB of each animal was estimated by applying the following formula:


$$N=\frac{Q\cdot V_{r\,e\,f}}{V_{d\,i\,s}}$$

We also compared whether the volumes of the different regions were affected by the surgical procedure. For this purpose, we calculated the index V/HL (volume/head length) to adjust the volume of the different regions according to the size of the animal and to be able to compare them with each other.

### 2.11. Statistical analyses

Animals were randomly assigned to each experimental group. We first conducted an exploratory graphical analysis of the data using boxplots (Quinn and Keough, 2002). Where parametric tests seemed indicated, the data were tested for normality and homoscedasticity with the Kolmogorov-Smirnov test and the *F*-test, respectively. Comparisons of proliferative activity between sulcal and intersulcal regions and between precommisural and commissural areas were assessed by parametric paired two-tailed *t*-tests. Comparison of proliferative activity among different regions was assessed by non-parametric Friedman’s test for repeated measures followed by Dunn’s *post-hoc* test for multiple comparisons. Effects of experimental surgery on neuron incorporation to the OB were assessed by independent samples *t*-tests. The level of significance was set at *p* < 0.05. Data are presented as mean ± SEM unless otherwise indicated. Statistical analyses were performed using GraphPad Prism version 9.4.1 for macOS (GraphPad Software, San Diego, California USA).^1^

## 3. Results

### 3.1. Cell composition and organization of the ventricular zone of *Podarcis liolepis*

The walls of the LVs of *P. liolepis* are lined by two types of radial glia cells: uniciliated (type B) and multiciliated (type E) cells. The distribution of these cell types along the ventricular walls has not yet been described in detail. Scanning electron microscopy of the surface of the ventricle revealed mainly uniciliated cells (i.e., type B cells) with only a small fraction of type E cells found in isolation or forming small clusters of 2–4 cells in the rostral wall of the lateral ventricle (Figure 1C). In contrast, we found a high density of type E cells in some specific regions, such as the VZ located between the sulcus ventralis and the sulcus terminalis (Figure 1D). Other areas were somewhat intermediate, with groups of 8–15 type E cells close to each other, but without forming a continuum (Figure 1E). This intermediate pattern is more prevalent at caudal levels of the cortex and in the septum.

### 3.2. Proliferating precursors are heterogeneously distributed in different telencephalic regions

In animals with a survival time of 1.5 h after [^3^H]-thymidine administration, most labeled cells were found in the walls of the LVs in direct contact with the ventricular lumen (Figure 1A). [^3^H]-thy^+^ cells were found in different telencephalic regions of the LVs, both in sulci and intersulcal regions (Figure 1B). When we analyzed their distribution, we found that proliferating cells were more frequent in sulcal regions (8.35 ± 1.48 [^3^H]-thy^+^ cells/1000 cells) than in intersulcal regions (3.26 ± 0.85 [^3^H]-thy^+^ cells/1000 cells; *p* = 0.025, two-tailed paired *t*-test, Figure 1F).

We next compared the proliferative activity of different sulcal and intersulcal regions. In agreement with the previous analysis (Figure 1F), the sulcus septomedialis had the highest density of proliferating cells (10.75 ± 1.89 [^3^H]-thy^+^ cells/1000 cells) followed by the sulcus ventralis/sulcus terminalis (sv/st; 8.81 ± 1.68 [^3^H]-thy^+^ cells/1000 cells). Both regions presented significant differences with the intersulcus corticalis, which was the region with the lowest density of proliferating cells among the studied areas (1.43 ± 0.46 [^3^H]-thy^+^ cells/1000 cells; Friedman’s test followed by Dunn’s *post-hoc* test, *p* = 0.012) (Figure 1G).

To assess whether the walls of the LV had different proliferative activity at different antero-posterior levels, we analyzed separately the same regions in an anterior telencephalic (pre-commissural) and a posterior (post-commissural) level. Proliferating cells were equally distributed in anterior and posterior levels in all regions except in the sulci ventralis/terminalis (Figure 1H). In this region a higher concentration of proliferating cells were found in the most anterior part (11.77 ± 2.00 [^3^H]-thy^+^ cells/1000 cells), a density similar to that found in the sulcus septomedialis, whereas at caudal levels the proliferative activity dropped dramatically (2.18 ± 1.23 [^3^H]-thy^+^cells/1000 cells; two-tailed paired *t*-test, *p* = 0.008) (Figure 1H).

These results show that proliferating cells are not homogeneously distributed along the walls of the LV, but rather aggregate in sulcal regions.

### 3.3. Neural progenitor cells and neuroblast identity

**FIGURE 2:**
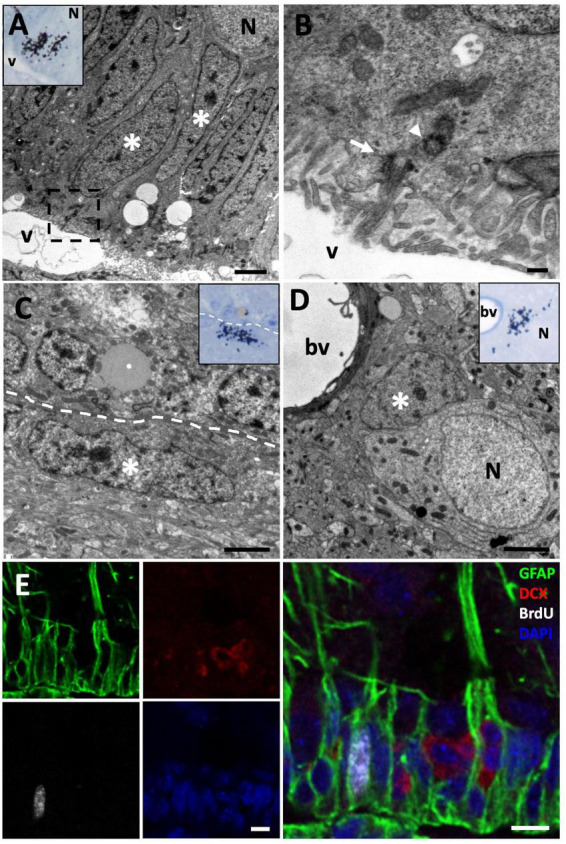
[^3^H]-thymidine-incorporating type B cells but not neuroblasts are found in the ventricular zone (VZ) at short survival times (1.5 h). **(A)** [^3^H]-thymidine-positive ([^3^H]-thy^+^) radial glia cells (asterisks) were identified in the sulcus medialis of the (VZ) in animals with survival times of 1.5 h. **(B)** Detail of the inset in the previous image, showing the contact of this type B cell with the ventricular lumen and the presence of a primary cilium (arrow) and its associated daughter centriole (arrowhead). **(C)** [^3^H]-thy^+^ B cell after 1.5 h survival in an intersulcal region. These cells generally present a flattened morphology. **(D)** [^3^H]-thy^+^ cell with glial features located in the brain parenchyma. **(E)** Immunofluorescence detection in the anterior olfactory nucleus of an animal injected with bromodeoxyuridine (BrdU) with a survival time of 1.5 h, in which BrdU^+^/GFAP^+^ cells are found, whereas all DCX^+^ neuroblasts at this time-point were BrdU^–^. bv, blood vessel; N, neuron; v, ventricle. Scale bars: **(A,C,D)** 2 μm; **(B)** 200 nm; **(E)** 5 μm.

In order to determine the identity and dynamics of neural progenitor cells in the LV of *P. liolepis* we administered [^3^H]-thymidine or BrdU, two proliferation markers, to different groups of lizards, and euthanized these specimens at different survival times (1.5, 6, 12, 24, and 72 h). In the group of lizards with a 1.5 h survival time, we found that all [^3^H]-thy^+^ cells corresponded to radial glia cells characterized by a single primary cilium in contact with the LV lumen, i.e., type B cells (Figure 2A). The primary cilium of B cells is characterized by the presence of a daughter centriole in perpendicular position to their basal body (Figure 2B). This is in sharp contrast to the cilia of E cells, which lack this centriole. Labeled B cells were normally isolated, although a few doublets were occasionally observed (Figure 4C). No labeled mitotic cells were found in this 1.5-h survival group. Comparing [^3^H]-thy^+^ cells located in sulcal and intersulcal regions, we observed that they were ultrastructurally very similar, the main difference between them being their cell shape. In the sulcal regions [^3^H]-thy^+^ cells had columnar morphology (Figure 2A), while in the intersulcal regions they were cuboidal or flattened cells (Figure 2C). We occasionally observed some [^3^H]-thy^+^ cells in the telencephalic brain parenchyma (Figure 2D). These were usually isolated, generally associated with large neurons. Their ultrastructure was characterized by scant cytoplasm and irregular nuclei with highly condensed chromatin, suggesting a glial identity.

**FIGURE 3:**
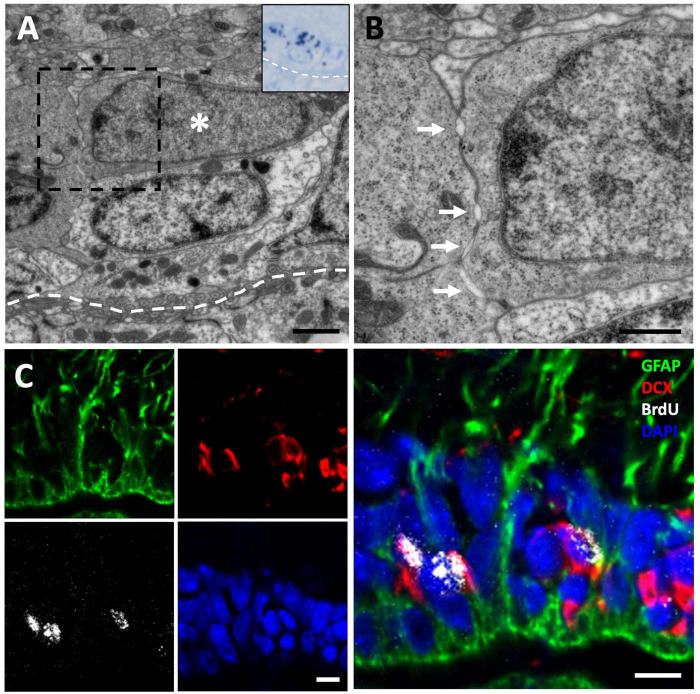
[^3^H]-thymidine-incorporating neuroblasts are found in the ventricular zone (VZ) at 3-day of survival. **(A)** [^3^H]-thymidine-positive ([^3^H]-thy^+^) neuroblast after 3 days of survival located in the VZ of the sulcus ventralis/terminalis. **(B)** Inset showing a detail of the previous cell, presenting typical ultrastructural features of migrating new neurons: scant cytoplasm with few organelles, high density of free ribosomes, and abundant intercellular spaces (arrows). **(C)** Immunofluorescence detection in the anterior olfactory nucleus of an animal injected with bromodeoxyuridine (BrdU) with a survival time of 3 days, in which BrdU^+^/DCX^+^ neuroblasts can be observed. Scale bars: **(A)** 1 μm; **(B)** 500 μm; **(C)** 5 μm.

To further characterize the differentiation and dynamics of proliferating cells, we studied longer survival times. In the group of lizards with 3 days of survival after [^3^H]-thymidine administration, we found that most [^3^H]-thy^+^ cells were still located in the VZ of the LVs. In these cases, we observed that most of them were typical B cells, although we also found cells presenting relaxed chromatin and scant cytoplasm with abundant ribosomes that did not contact the ventricle (Figure 3A). These cells also presented intercellular spaces, typical of migrating cells, thus suggesting that they corresponded to type A cells or neuroblasts (Figure 3B).

**FIGURE 4:**
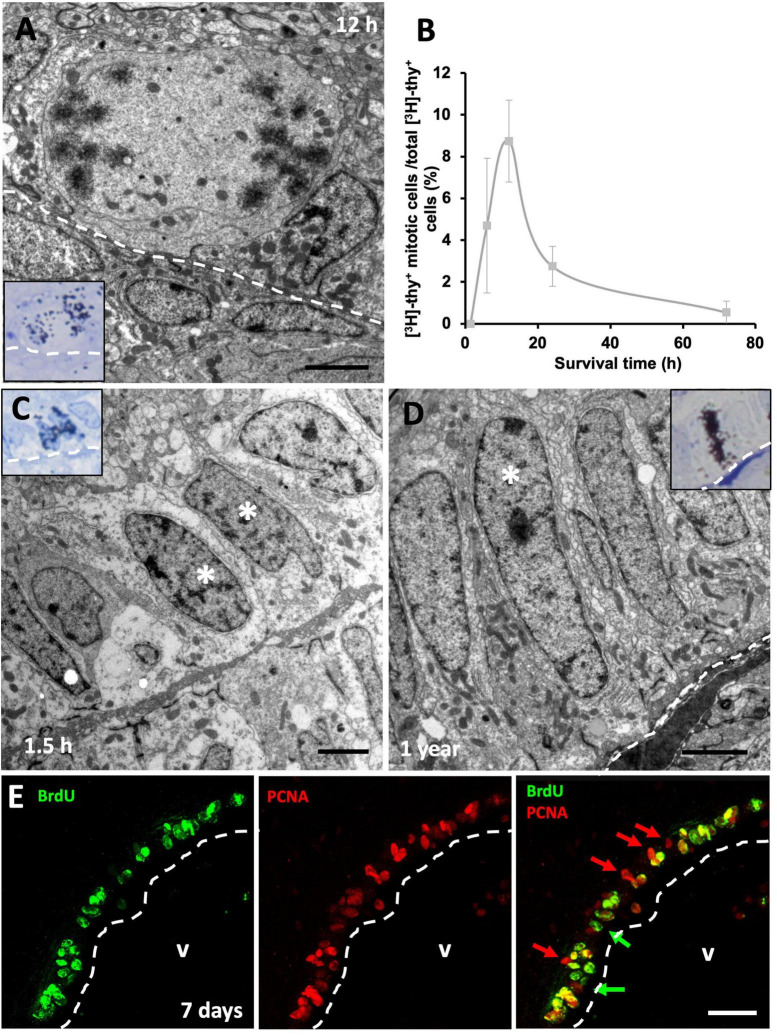
Proliferative rate of neural progenitors in the ventricular zone (VZ). **(A)** [^3^H]-thymidine-labeled ([^3^H]-thy^+^) mitotic cells in contact with the lateral ventricle (LV) lumen were quantified 12 h following [^3^H]-thymidine administration. **(B)** Quantification of the percentage of [^3^H]-thy^+^ cells in mitosis relative to all [^3^H]-thy^+^ cells in the VZ 1.5, 6, 12, 24, and 72 h after [^3^H]-thymidine administration. **(C)** Doublet of non-mitotic [^3^H]-thy^+^ cells (asterisks) in the 1.5 h survival group. **(D)** Slow-dividing [^3^H]-thy^+^ type B cell (asterisk), corresponding to a uniciliated radial glia in contact with the LV lumen, 1 year after [^3^H]-thymidine administration. **(E)** BrdU/PCNA immunofluorescence detection in the sulcus medialis of an animal injected with BrdU with a survival time of 7 days. Most cells are double-positive, although some BrdU^–^/PCNA^+^ and some BrdU^+^/PCNA^–^ cells were also detected. Scale bars: **(A–C)** 2 μm; **(E)** 25 μm.

In order to confirm our results about the cell identity of [^3^H]-thy^+^ cells, we performed immunohistochemistry detection of a radial glia marker, glial fibrillary acidic protein (GFAP), and a neuroblast marker, doublecortin (DCX), combined with the proliferation marker BrdU after 1.5 h or 3 days of survival. In the group with a 1.5 h survival time after BrdU administration, we observed that virtually all BrdU positive (BrdU^+^) cells were located in the walls of the LVs and were GFAP positive (GFAP^+^) with radial processes, confirming our previous results and thus strongly suggesting that they were radial glia (Figure 2E). When the survival time after BrdU administration was extended to 3 days, we found that in addition to double BrdU^+^/GFAP^+^ labeled cells in contact with the ventricle, there was also a population of double BrdU^+^/DCX^+^ cells that did not contact the ventricle, thus suggesting that they corresponded to migrating new neurons or neuroblasts (Figure 3C).

### 3.4. Fast- and slow-dividing progenitor cells are present in the VZ

We next assessed cell cycle dynamics in VZ progenitors. To that purpose, we performed a time-lapse with different survival times (1.5, 6, 12, 24, and 72 h) after [^3^H]-thymidine administration, which allowed us to study how long it took the proliferating cells to enter mitosis after DNA duplication (S phase). As expected, we found that none of the [^3^H]-thy^+^ cells were undergoing mitosis 1.5 h after [^3^H]-thymidine administration, suggesting that longer periods of time are needed by neural precursors to transition from S to M phase. When we examined specimens with longer survival times, we found [^3^H]-thy^+^ mitotic cells as soon as 6 h after administration, although they peaked at 12 h of survival time (Figures 4A, B). After that, the percentage of [^3^H]-thy^+^ mitotic cells began to decrease in the 24 h-survival group, being practically non-existent at 72 h (Figure 4B). In all cases, mitotic cells were in contact with the ventricular lumen (Figure 4A). We occasionally found non-mitotic [^3^H]-thy^+^ cells forming doublets (Figure 4C), but the fact that we found these cells even at the shortest survival time-point (1.5 h), suggests that these doublets correspond to adjacent precursors that entered cell cycle simultaneously rather than to two daughter cells resulting from the same mitotic event.

With longer survival times (1, 3, 6, and 12 months) after [^3^H]-thymidine administration we found type B and some type A [^3^H]-thy^+^ cells in the VZ of the lateral ventricles (Figure 4D). Some cells showed strong labeling, while others were somewhat weaker, suggesting that several preceding mitotic events occurred during the survival time between the administration of the proliferation marker and euthanasia. In none of the cases studied labeled type E cells were detected.

By combining two proliferation markers, BrdU and PCNA, we expected that those cells that were doubly-labeled would correspond to those which have undergone at least two rounds of cell division. In all telencephalic levels analyzed, most cells were found to be BrdU^+^/PCNA^+^ doubly-labeled, although single-labeled BrdU^+^/PCNA^–^ and BrdU^–^/PCNA^+^ cells were also found (Figure 4E). Together, the results of these experiments suggest that two subpopulations of neural precursor cells are present in the VZ of the lizard *P. liolepis*, one that is actively and continuously dividing, and another that is more quiescent or slow-dividing.

### 3.5. Neuroblasts tangentially migrate from the VZ toward the OB

**FIGURE 5:**
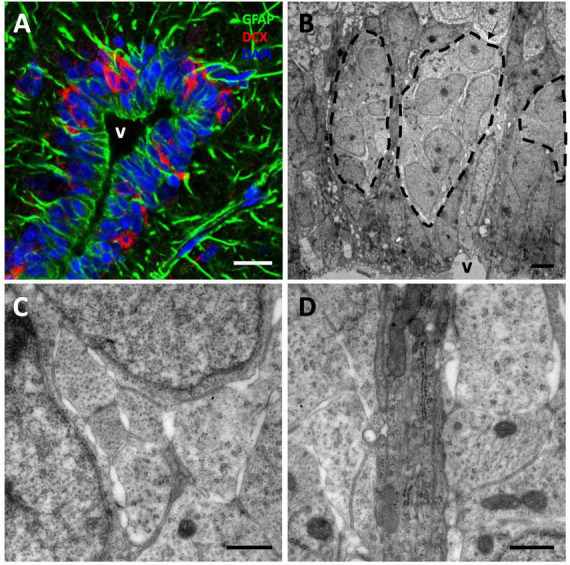
Tangential migration of neuroblasts in the anterior olfactory nucleus (AON). **(A)** GFAP/DCX immunofluorescence image showing clusters of DCX+ neuroblasts intermingled with GFAP^+^ radial glia processes. **(B)** Transmission electron microscopy image of neuroblast clusters surrounded by radial glia fibers. **(C)** Detail of the spaces left by the neuroblasts between each other allowing their migration. **(D)** High magnification image showing a detail of several radial glia fibers, darker, between more electron-lucent neuroblast processes. Scale bars: **(A)** 10 μm; **(B)** 2 μm; **(C,D)** 500 nm.

We next asked whether a tangential migration of new neurons exists from the VZ to the OBs of *P. liolepis*. In our ultrastructural analysis of the VZ surface we identified putative chains of neuroblasts migrating longitudinally, just adjacent to radial glia cells. We found this type of migrating chains at anterior levels of the sulcus ventralis/sulcus terminalis, in the AON and in the olfactory peduncle, suggesting a tangential migration toward the OBs. These migrating cell clusters were in contact with radial glia cells, partly wrapped by their fibers, as evidenced by our immunofluorescence detection of DCX and GFAP (Figure 5A). Nevertheless, further ultrastructural examination suggested that migrating new neurons were not totally isolated, but they were in contact with the surrounding brain parenchyma or even with mature neurons located dorsally to the VZ (Figure 5B). Detailed examination of these migrating cells revealed that they had nuclei with relaxed chromatin and a prominent nucleolus, and scant cytoplasm with abundant microtubules and ribosomes (Figures 5B, C). Abundant free spaces were also observed between these cells, a distinctive characteristic of migrating cells, and they were in close contact with radial glia fibers (Figure 5D).

**FIGURE 6:**
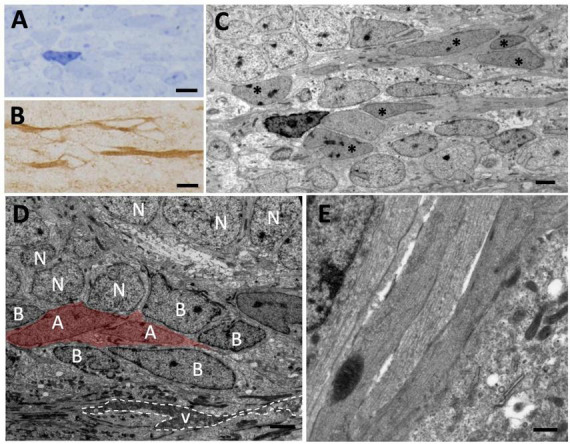
Tangential migration of neuroblasts in the olfactory peduncle. **(A)** Longitudinal section of the olfactory peduncle stained with toluidine blue. **(B)** Consecutive section to that shown in panel **(A)** on which post-embedding immunohistochemistry for DCX was performed. Several DCX^+^ cells with elongated morphology can be observed. **(C)** Transmission electron microscopy (TEM) image of the region shown in panel **(A)**. The DCX^+^ cells shown in panel **(B)** can be observed (asterisks). **(D)** Neuroblasts (with their nuclei labeled with an A) migrate over radial glia cells (labeled with B) that line the ventricle of the olfactory peduncle and are also in direct contact with adjacent neurons (N). **(E)** Detail of several neuroblasts processes sectioned longitudinally with presence of microtubules. Scale bars: **(A,B)** 5 μm; **(C,D)** 2 μm; **(E)** 500 nm.

To further investigate this population of migrating DCX^+^ cells, we next performed a sagittal view analysis of the olfactory peduncle in animals injected with [^3^H]-thymidine and with survival times of 1.5 and 72 h. We detected chains of spindle-shaped [^3^H]-thy^+^ cells with oval nuclei, relaxed chromatin and large nucleoli (Figures 6A–D) which, after correlating with the immunostained sections, were found to be DCX^+^/GFAP^–^ cells (Figures 6A–C). These cells had long cytoplasmic expansions rich in microtubules and presented the characteristic intercellular gaps of migrating cells (Figure 6E). Together, these observations indicate the existence of a tangential migration of new neurons from the VZ toward the OBs of *P. liolepis*.

### 3.6. Tangential migration of neuroblasts contributes to neuron incorporation in the OB

**FIGURE 7:**
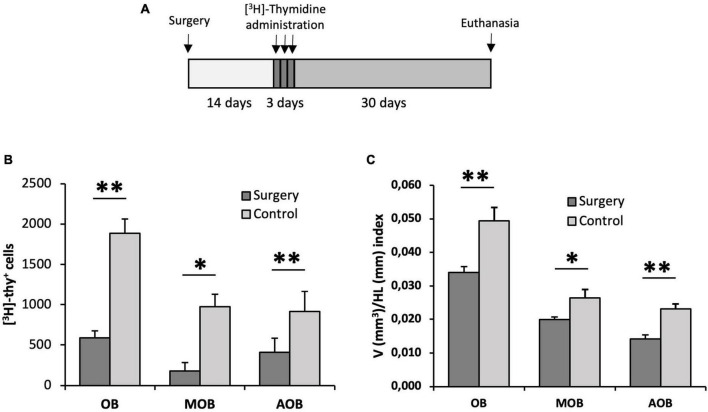
Proliferation assessment in the olfactory bulb of animals with their olfactory peduncle sectioned. **(A)** Surgery and [^3^H]-thymidine administration protocol. **(B)** Quantification of the total number of [^3^H]-thymidine-positive cells found in the olfactory bulb (OB), main olfactory bulb (MOB) and accessory olfactory bulb (AOB) using the physical dissector. Cell proliferation in all regions was significantly reduced in lizards with their olfactory peduncles sectioned compared with control animals (*n* = 5, two-tailed unpaired *t*-test). **(C)** Cavalieri estimation of the volume of the OB, MOB, and AOB relative to the head length in the surgery and control groups. This volume/head length index was significantly lower in the surgery group (*n* = 5, two-tailed unpaired *t*-test). Data are represented as the mean ± SEM. *p < 0.05, ^**^p < 0.01.

After describing a stream of neuroblasts migrating from anterior levels of the sv/st to the OB, we asked whether these cells were, at least in part, responsible for the adult neurogenesis previously observed in the OB of lizards. To this end, we performed an experiment with two groups of lizards. In one group, we surgically excised the OB from the rest of the telencephalon by sectioning at the level of the olfactory peduncle, thus preventing any cells from the anterior telencephalic levels from reaching the OB. In a second group of animals (control group), we performed an identical procedure except for the fact that the olfactory peduncle was not excised and remained intact. After 2 weeks post-surgery, lizards were administered [^3^H]-thymidine and allowed to survive 1 month (Figure 7A). We then performed unbiased stereological quantification of the number of [^3^H]-thy^+^ cells using the physical dissector method. Using this approach, we found a significantly higher number of [^3^H]-thy^+^ cells in the OB of the control group than in the lizards subjected to surgery (1,888.89 ± 250.02 vs. 589.58 ± 173.52 [^3^H]-thy^+^ cells, *p* = 0.007, two-tailed independent samples *t*-test) (Figure 7B). We further investigated whether there were differences between the MOB and the AOB. Our results indicate that the same trend observed for the whole OB holds for both the MOB and the AOB separately, with a significantly higher number of [^3^H]-thy^+^ cells in the control group than in the lizards subjected to surgery (MOB: control 975.00 ± 175.66 vs. surgery group 179.17 ± 83.51 [^3^H]-thy^+^ cells, *p* = 0.006, two-tailed independent samples *t*-test; AOB: control 913.89 ± 153.68 vs. surgery group 410.42 ± 104.38 [^3^H]-thy^+^ cells, *p* = 0.037, two-tailed independent samples *t*-test) (Figure 7B).

Based on the significantly higher proliferative activity in the OB of control animals compared to those subjected to surgery, we wondered whether these differences might have an impact on the whole volume of the OB. To test this hypothesis, we measured the volume of the OBs relative to the size of the head using the Cavalieri method, thus obtaining a volume/head length index (V/HL index). We found that the animals in the control group had a larger V/HL index than those with their olfactory peduncle excised (0.049 ± 0.004 vs. 0.034 ± 0.002, respectively, *p* = 0.008, two-tailed independent samples *t*-test). Furthermore, when we compared this index separately in the MOB and AOB we observed that in both cases it was significantly higher in the control group than in the surgery group but, interestingly, this difference was more pronounced in the AOB than in the MOB (MOB: control 0.026 ± 0.003 vs. surgery 0.020 ± 0.001, *p* = 0.049, two-tailed independent samples *t*-test; AOB: control 0.023 ± 0.001 vs. surgery 0.014 ± 0.001, *p* = 0.002, two-tailed independent samples *t*-test) (Figure 7C).

### 3.7. Neuronal maturation in the medial cortex

**FIGURE 8:**
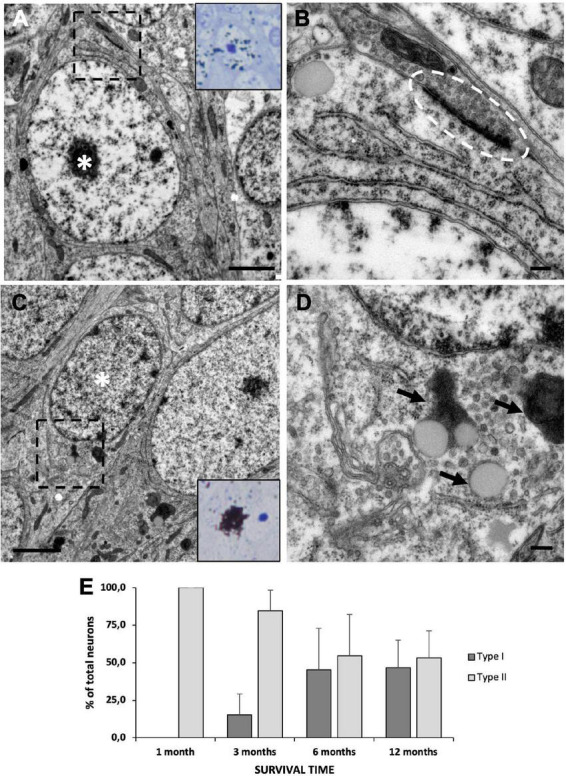
Neuronal maturation in the medial cortex (MC). **(A)** MC neuron labeled with [^3^H]-thymidine after 1 month of survival following administration (asterisk). It presents large nucleus with relaxed chromatin and a prominent nucleolus, and electron-dense cytoplasm with abundant free ribosomes, typical features of type II neurons. **(B)** Detail of a synapsis (dotted oval) established by a type II neuron. **(C)** MC neuron labeled with [^3^H]-thymidine after 1 year of survival following administration (asterisk). It presents a small nucleus with clumped chromatin and electron-lucent cytoplasm, typical of type I neurons. **(D)** Detail of lipofuscin aggregates (arrows) in a type I neuron. **(E)** Quantification of the percentage of type I and type II [^3^H]-thymidine-positive neurons at different survival times after administration. In the MC, the percentage of type I neurons increases with time, while that of type II neurons decreases accordingly. Data are represented as the mean ± SEM. Scale bars: **(A,C)** 2 μm; **(B,D)** 200 nm.

After [^3^H]-thymidine administration, we assessed whether the new neurons migrating to the MC preferentially contributed to the generation of type I, type II, or both neuronal types at different survival times (1, 3, 6, and 12 months). Our ultrastructural analysis revealed that after 1 month of survival, only type II neurons could be found labeled with [^3^H]-thymidine (Figure 8A). These cells had established synapses with neighboring neurons, hence suggesting that they were functionally mature 1 month after they were generated (Figure 8B). In the MC of the groups with longer survival times, we found that both [^3^H]-thy^+^ type I (Figure 8C) and [^3^H]-thy^+^ type II neurons could be found from 3 months onward. Type I neurons could be easily identified by the presence of clumped chromatin, lipofuscin cytoplasmic aggregates, and scarce organelles (Figure 8D). We quantified the percentage of [^3^H]-thy^+^ type I neurons across the different survival times and found an increasing trend with survival time, going from 15.4 ± 13.8% after 3 months of survival to 46.8 ± 18.2% after 12 months (Figure 8E). Conversely, and coincident with the increase in type I neurons, the percentage of type II neurons decreased with increasing survival times, suggesting the intriguing possibility that a subset of type II neurons might be maturing to form type I neurons.

## 4. Discussion

### 4.1. Neural stem cell identity and distribution in the VZ of *Podarcis liolepis*

The incorporation of new neurons into preexisting neural networks requires the precise coordination of several processes, including cell proliferation, migration, and integration into adult neural circuits (Paredes et al., 2016). The VZ of the lateral ventricles is the most phylogenetically conserved neurogenic niche among the different vertebrate groups, including reptiles (González-Granero et al., 2008). The cell composition and organization of the VZ differs across vertebrate groups. In reptiles, birds and mammals, the VZ is composed of uni- and multiciliated cells in contact with the ventricular lumen, which are known as B and E cells, respectively. In the case of birds, uniciliated and multiciliated cells are heterogeneously distributed (García-Verdugo et al., 2002). In this study, we show that this disparate distribution of radial glia cells surrounding the VZ is also present in the lizard *P. liolepis*. We observed that in most of the VZ surface of this species B cells predominate over E cells except for some specific areas, such as the region between the sulcus ventralis and the sulcus terminalis, in which we found the reverse situation.

Our results from proliferation studies with [^3^H]-thymidine indicate that the neural stem cells responsible for adult neurogenesis in lizards are type B cells, i.e., uniciliated radial glia, GFAP-positive cells with an apical contact with the ventricular lumen. This is in agreement with the data obtained on the identity of neural stem cells in birds and mammals (García-Verdugo et al., 2002).

In birds, the primary neural progenitors are cells with similar characteristics to those of reptiles: uniciliated cells with radial glia characteristics, in contact with the ventricle and GFAP-positive (Alvarez-Buylla et al., 1998). The similarity in neural stem cell features between reptiles and birds contrasts with those of adult mammals. In mammals, the neural stem cells belong to the astroglial lineage, in both the ventricular-subventricular zone (V-SVZ) and the subgranular layer of the hippocampal dentate fascia. These cells are also known as type B cells, and they are characterized by GFAP expression. Nevertheless, they lack long radial expansions and normally only contact the LV with their single primary cilium (Doetsch et al., 1999). However, when analyzed in embryonic and early postnatal stages, these cells expose large surfaces to the ventricular lumen, which are progressively reduced until they are practically nonexistent in the adult (Tramontin et al., 2003; Merkle et al., 2004). Thus, considering their characteristics in perinatal stages, mammalian neural stem cells are not so unlike those of sauropsids (birds and reptiles).

The presence of an additional cell type labeled with [^3^H]-thymidine in lizards with a survival time of 3 days compared to those with short survival times (i.e., 1.5 h), suggests that this cell type could correspond to newly formed neuroblasts (or type A cells). This was supported by the ultrastructural characteristics of these cells, which were very similar to those of the migratory cells previously described in the internal plexiform layer of the MC of *P. liolepis* (Garcia-Verdugo et al., 1986). Newly formed neuroblasts were very similar to those described in birds and mammals (Alvarez-Buylla et al., 1998). However, unlike what it is reported in mammals (Doetsch et al., 1997), there is no evidence that in lizards these cells have proliferative activity, since at short survival times no [^3^H]-thy^+^ type A cells were found. As new neurons migrate, they undergo changes in their nuclei, such as an increase in their volume and chromatin condensation, accompanied by an enlargement of the rough endoplasmic reticulum and Golgi apparatus. These morphological changes seem to indicate that, during the course of migration, neuroblasts differentiate and acquire a mature neuronal phenotype (Garcia-Verdugo et al., 1986).

### 4.2. Neural stem cell proliferative activity

The heterogeneous distribution of type B and type E cells in the walls of the VZ prompted us to study whether cell proliferation was concentrated in any specific regions. The results of our proliferation analysis indicate that proliferative cells are more abundant in sulcal areas compared to intersulcal regions. This finding was previously observed in other reptile species such as the lizard *Tarentola mauritanica* and *Eublepharis macularius*, while in others, such as in the turtle *Trachemys scripta*, a clear correspondence has not been established (Pérez-Cañellas et al., 1997; Font et al., 2001; McDonald and Vickaryous, 2018). The concentration of proliferating cells in certain regions of the VZ resembles the so-called hot-spots described in birds (Alvarez-Buylla et al., 1990).

Based on the labeling pattern obtained with our time-lapse survival experiment after [^3^H]-thymidine administration and double immunodetection of BrdU and PCNA, it seems that most neural progenitors have a rapid proliferation rate, dividing at least two times in an interval of 3–7 days. However, it is worth noting that labeled cells are also present in the VZ of specimens with 1 year of survival after [^3^H]-thymidine administration. These cells have morphological and ultrastructural characteristics of type B cells. The fact that the label has not yet been diluted in these cells indicates that their proliferation rate was very low during that period. Therefore, we hypothesize that there might be at least two subpopulations of type B cells, including a fast- and a slow-dividing population. This possibility is consistent with the presence of two similar actively dividing and quiescent subpopulations of neural stem cells in the brain of mammals (Pastrana et al., 2009; Codega et al., 2014; Cebrián-Silla et al., 2017). In any case, it must be considered that the animals used in this study were maintained in captivity, a fact that could perhaps influence the rate of neurogenesis and thus explain the presence of slow-dividing progenitors (Delgado-González et al., 2008). On the other hand, it has been suggested that proliferation may be subjected to seasonal fluctuations, although the evidence is controversial. Delgado-González et al. (2008), Delgado-Gonzalez et al. (2011) reported seasonal differences in the number of BrdU-labeled cells in chemosensory brain areas of *Gallotia galloti* lizards. However, Sampedro et al. (2008), using similar techniques, did not detect significant seasonal changes in size or proliferative activity in these same areas in *P. liolepis*.

### 4.3. Neuroblast migration and differentiation in the olfactory bulb

Among the different regions studied, the sulcus ventralis/terminalis shows a relatively high density of proliferative cells at anterior brain levels. However, the number of new neurons incorporated into the adjacent striatum is rather low (Font et al., 2001). This led us to hypothesize that the new neurons generated in this region might migrate to some distant target location. In addition, when studying the ultrastructure of this area we found clusters of migrating cells transversely sectioned, indicating that they might be involved in a longitudinal migration parallel to the surface of the LV. This type of migration to the OB has previously been described in other reptile and mammal species (Lois and Alvarez-Buylla, 1994; Pérez-Cañellas and García-Verdugo, 1996; de Pimentel et al., 2021). As a result, we hypothesized that the OB was one of the candidate destinations for these cells, which we tested by surgically sectioning the olfactory peduncles in a group of animals. The lower number of [^3^H]-thy^+^ cells in the OB after the physical excision of the olfactory peduncles, together with a reduced OB volume suggests that the new neurons incorporated to this region are not exclusively generated *in situ* around the OB ventricle, but also come from more caudal levels. We propose that the sulci ventralis/terminalis is the origin of part of the new neurons incorporated into the OB of *P. liolepis* by tangential migration. Therefore, a non-negligible fraction of new OB neurons would be born in the telencephalic VZ, caudal to the bulbs, and then migrate long distances until they reach their destination (Peñafiel et al., 1996). In their migration, the cells seem to follow specific routes restricted to the vicinity of the VZ (Font et al., 2001). These long migrations toward the OB are also observed in mammals, where neuroblasts follow a migration route called rostral migratory stream (Lois and Alvarez-Buylla, 1994). Nevertheless, in contrast with mammals, in which migrating new neurons are isolated from the rest of the brain parenchyma by gliotubes formed by astrocytes (Lois et al., 1996), similar isolation mechanisms have not been observed in *P. liolepis*, nor in other reptile species.

### 4.4. Neuronal maturation in the medial cortex

The MC is homologous to the hippocampal dentate gyrus of mammals (Olucha et al., 1988; Medina et al., 2017; Desfilis et al., 2018), and harbors most of adult neurogenesis in the brain of lizards (Font et al., 2001). We studied newly generated neurons in the MC by monitoring their maturation at different times after the administration of [^3^H]-thymidine, from 1 to 12 months. Most of adult-born neurons of *P. liolepis* are incorporated into the MC cell layer, which contains at least two types of neurons based on the ultrastructure of their soma. Notably, only type II neurons were labeled with [^3^H]-thymidine at the shortest survival time (1 month). However, as survival time increased, the number of [^3^H]-thy^+^ type I neurons also increased. Therefore, we surmise that type II neurons are young cells generated from neuronal precursors, and through a maturation process they transform into type I neurons. This would explain the different proportion of the two types of neurons in lizards of different ages, while in newborns all neurons are type II, in adult animals type I neurons are predominant (López-García et al., 1984). Interestingly, the presence of a prominent nucleolus, relaxed chromatin, and high number of ribosomes in young cells compared to mature cells would indicate a higher gene expression and protein production in the former and a higher degree of specialization in the second. Few studies have examined the maturation of adult-born neurons beyond 4–6 weeks and none has investigated the ultrastructural changes during maturation. However, Cole et al. (2020) recently studied changes in several morphological features of rat hippocampal adult-born neurons and found that they continue to change their morphology over 24 weeks after birth. In our study the highest number of type I neurons was observed at 12 months of maturation. The great heterogeneity of the main neurons of the lizard MC with respect to soma size, dendritic tree pattern and spine density (Luis de la Iglesia and Lopez-Garcia, 1997) could be the consequence of this long maturation process. In the future, it would be very interesting to address the study of this prolonged maturation process in the cerebral cortex of *P. liolepis* by studying morphological developmental changes of retrovirus-labeled neurons.

The fact that at all survival times we find [^3^H]-thy^+^ type II neurons is most likely due to continuous production of these neurons from type B type cells. Indeed, [^3^H]-thy^+^ type B cells were still found in the VZ of all specimens even with the longest survival time (1 year), most probably corresponding to cells with a slower proliferation rate that still retained [^3^H]-thymidine labeling.

**FIGURE 9:**
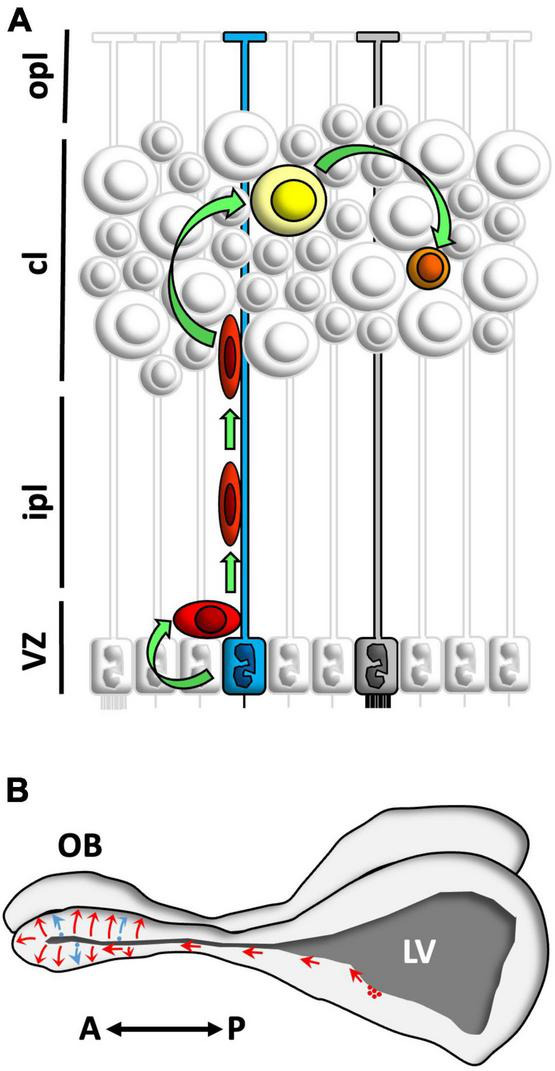
Schematic summary of the main findings. **(A)** Neural stem cells in the ventricular zone (VZ) of *Podarcis liolepis* were identified as uniciliated radial glia cells in contact with the ventricular lumen (blue). These cells proliferate and give rise to neuroblasts (red) which migrate and incorporate in different telencephalic regions such as the medial cortex (MC) or the olfactory bulb (OB). In the MC, neuroblasts differentiate into type II neurons (yellow), which eventually mature to form type I neurons (represented in orange). In the VZ there are also multiciliated radial glia cells (gray), which do not proliferate. **(B)** Neuroblasts generated in the anterior levels of the sulcus ventralis/sulcus terminalis of *P. liolepis* (red) migrate tangentially and reach the OB, where they disperse radially, differentiate, and integrate into pre-existing neural circuits. Furthermore, there is a small part of the new neurons that is generated *in situ* in the OB ventricles (blue). A, anterior; cl, cell layer; ipl, inner plexiform layer; LV, lateral ventricle; opl, outer plexiform layer; P, posterior; sv/st, sulcus ventralis/sulcus terminalis; VZ, ventricular zone.

In summary, this study provides evidence about neural stem cell identity, routes of neuroblast migration, and neuronal maturation in the telencephalon of the lizard *P. liolepis* (Figure 9). Our results are generally in agreement with studies of adult neurogenesis in other lacertids and in a few lizard species outside of Lacertidae (*Anolis carolinensis*, Duffy et al., 1990; *Tarentola mauritanica*, Pérez-Cañellas and García-Verdugo, 1996; *Tropidurus hispidus*, Marchioro et al., 2005; *Phrynocephalus vlangalii*, Shao et al., 2012; *Eublepharis macularius*, McDonald and Vickaryous, 2018). These findings suggest that adult neurogenesis may be an evolutionarily conserved and taxonomically widespread phenomenon among lizards. However, given the sheer abundance and diversity of living lizards, it seems premature to extrapolate results obtained from work with a handful of species to the group as a whole. More studies are clearly needed to properly characterize adult neurogenesis and related phenomena (e.g., regeneration) in lizards and in other reptiles.

Adult neurogenesis and brain regeneration in reptiles have been known for several decades, yet they have been largely ignored by neurobiologists interested in neurogenesis and brain development. Research in this area is still dominated by anthropocentric (or at least mammalocentric) thinking. However, their crucial phylogenetic position makes reptiles, and lizards in particular, key actors in the neurogenesis play (Jacyniak et al., 2017). The similarities and differences of adult neurogenesis in lizards and in other, more intensively studied taxa, such as mammals and birds, underscore the need for the type of broad comparative studies advocated by Zupanc (2001) and others. Understanding the cellular processes promoting neural stem cell proliferation, migration and differentiation in lizards can offer insights into the widespread loss of adult neurogenesis and regenerative capabilities in mammals, and may allow the development of strategies to selectively target and modulate these processes in the adult brain.

## Data availability statement

The raw data supporting the conclusions of this article will be made available by the authors, without undue reservation.

## Ethics statement

This animal study was reviewed and approved by Valencian Council of Territory and Habitat (registry number: 2007/4900).

## Author contributions

SG-G, ED, EF, and JG-V conceived the original idea. SG-G and ED performed the experimental procedures under the supervision of JG-V. EF and JG-V provided experimental resources. SG-G, EF, VH-P, and JG-V participated in data visualization, formal analysis, and validation. SG-G, VH-P, and JG-V wrote the original draft. ED and EF provided manuscript draft revisions. VH-P and JG-V obtained funding for the study. All authors contributed to the article and approved the final manuscript.

## Footnotes

1
www.graphpad.com

## References

[B1] Altman J. Das G. D. (1965). Autoradiographic and histological evidence of postnatal hippocampal neurogenesis in rats. *J. Comp. Neurol.* 124 319–335. 10.1002/cne.901240303 5861717

[B2] Alvarez-Buylla A. García-Verdugo J. M. Mateo A. S. Merchant-Larios H. (1998). Primary neural precursors and intermitotic nuclear migration in the ventricular zone of adult canaries. *J. Neurosci.* 18 1020–1037. 10.1523/JNEUROSCI.18-03-01020.1998 9437023 PMC6792779

[B3] Alvarez-Buylla A. Theelen M. Nottebohm F. (1990). Proliferation “hot spots” in adult avian ventricular zone reveal radial cell division. *Neuron* 5 101–109. 10.1016/0896-6273(90)90038-H 2369518

[B4] Bayer S. A. (1983). 3H-thymidine-radiographic studies of neurogenesis in the rat olfactory bulb. *Exp. Brain Res.* 50 329–340. 10.1007/BF00239197 6641865

[B5] Birse S. C. Leonard R. B. Coggeshall R. E. (1980). Neuronal increase in various areas of the nervous system of the guppy. Lebistes. *J. Comp. Neurol.* 194 291–301. 10.1002/cne.901940202 7440802

[B6] Cebrián-Silla A. Alfaro-Cervelló C. Herranz-Pérez V. Kaneko N. Park D. H. Sawamoto K. (2017). Unique organization of the nuclear envelope in the post-natal quiescent neural stem cells. *Stem Cell Rep.* 9 203–216. 10.1016/j.stemcr.2017.05.024 28648897 PMC5511107

[B7] Codega P. Silva-Vargas V. Paul A. Maldonado-Soto A. R. DeLeo A. M. Pastrana E. (2014). Prospective identification and purification of quiescent adult neural stem cells from their in vivo niche. *Neuron* 82 545–559. 10.1016/j.neuron.2014.02.039 24811379 PMC4360885

[B8] Cole J. D. Espinueva D. F. Seib D. R. Ash A. M. Cooke M. B. Cahill S. P. (2020). Adult-Born hippocampal neurons undergo extended development and are morphologically distinct from neonatally-born neurons. *J. Neurosci.* 40 5740–5756. 10.1523/JNEUROSCI.1665-19.2020 32571837 PMC7380968

[B9] Crespo C. Gracia-Llanes F. J. Blasco-Ibáñez J. M. Gutièrrez-Mecinas M. Marqués-Marí A. I. Martínez-Guijarro F. J. (2003). Nitric oxide synthase containing periglomerular cells are GABAergic in the rat olfactory bulb. *Neurosci. Lett.* 349 151–154. 10.1016/S0304-3940(03)00819-X 12951191

[B10] Davila J. C. Guirado S. De La Calle A. Marin-Giron F. (1985). Electron microscopy of the medial cortex in the lizard *Psammodromus algirus*. *J. Morphol.* 185 327–338. 10.1002/jmor.1051850305 29969868

[B11] Delgado-González F. J. Alonso-Fuentes A. Delgado-Fumero A. García-Verdugo J. M. González-Granero S. Trujillo-Trujillo C. M. (2008). Seasonal differences in ventricular proliferation of adult *Gallotia galloti* lizards. *Brain Res.* 1191 39–46. 10.1016/j.brainres.2007.10.092 18178172

[B12] Delgado-Gonzalez F. J. Gonzalez-Granero S. Trujillo-Trujillo C. M. García-Verdugo J. M. Damas-Hernandez M. C. (2011). Study of adult neurogenesis in the *Gallotia galloti* lizard during different seasons. *Brain Res.* 1390 50–58. 10.1016/j.brainres.2011.03.027 21419108

[B13] Desfilis E. Abellán A. Sentandreu V. Medina L. (2018). Expression of regulatory genes in the embryonic brain of a lizard and implications for understanding pallial organization and evolution. *J. Comp. Neurol.* 526 166–202. 28891227 10.1002/cne.24329 PMC5765483

[B14] Doetsch F. Caillé I. Lim D. A. García-Verdugo J. M. Alvarez-Buylla A. (1999). Subventricular zone astrocytes are neural stem cells in the adult mammalian brain. *Cell* 97 703–716. 10.1016/S0092-8674(00)80783-7 10380923

[B15] Doetsch F. Garcća-Verdugo J. M. Alvarez-Buylla A. (1997). Cellular composition and three-dimensional organization of the subventricular germinal zone in the adult mammalian brain. *J. Neurosci.* 17 5046–5061. 10.1523/JNEUROSCI.17-13-05046.1997 9185542 PMC6573289

[B16] Duffy M. T. Simpson S. B. Jr. Liebich D. R. Davis B. M. (1990). Origin of spinal cord axons in the lizard regenerated tail: supernormal projections from local spinal neurons. *J. Comp. Neurol.* 293 208–222. 10.1002/cne.902930205 19189712

[B17] Easter S. S. (1983). Postnatal neurogenesis and changing connections. *Trends Neurosci.* 6 53–56. 10.1016/0166-2236(83)90025-5

[B18] Ebbesson S. O. E. Voneida T. J. (1969). The cytoarchitecture of the pallium in the tegu lizard (*Tupinambis nigropunctatus*). *Brain Behav. Evol.* 2 431–466. 10.1159/000125899

[B19] Eriksson P. S. Perfilieva E. Björk-Eriksson T. Alborn A. M. Nordborg C. Peterson D. A. (1998). Neurogenesis in the adult human hippocampus. *Nat. Med.* 4 1313–1317. 10.1038/3305 9809557

[B20] Font E. Desfilis E. Pérez-Cañellas M. M. Garcia-Verdugo J. M. (2001). Neurogenesis and neuronal regeneration in the adult reptilian brain. *Brain Behav. Evol.* 58 276–295. 10.1159/000057570 11978946

[B21] Font E. García-Verdugo J. M. Desfilis E. Pérez-Cañellas M. (1995). “Neuron—Glia interrelations during 3-Acetylpyridine-Induced degeneration and regeneration in the adult lizard brain,” in *Neuron—Glia Interrelations During Phylogeny: II. Plasticity and Regeneration*, eds Vernadakis A. Roots B. I. (Totowa, NJ: Humana Press), 275–302. 10.1007/978-1-59259-468-9_11

[B22] García Verdugo J. M. Berbel P. J. López García C. (1981). Golgi and electron microscopy study of cerebral ependymocytes of the lizard *Lacerta galloti*. *Trab. Inst. Cajal.* 72 269–278. 6192583

[B23] Garcia Verdugo J. M. Berbel Navarro P. Regidor Garcia J. Lopez Garcia C. (1984). Ultrastructure of neuronal cell bodies in the medial cortex of *Lacerta galloti*. *J. Hirnforsch.* 25 187–196. 6736634

[B24] Garcia-Verdugo J. M. Farinas I. Molowny A. Lopez-Garcia C. (1986). Ultrastructure of putative migrating cells in the cerebral cortex of *Lacerta galloti*. *J. Morphol.* 189 189–197. 10.1002/jmor.1051890209 3746917

[B25] García-Verdugo J. M. Ferrón S. Flames N. Collado L. Desfilis E. Font E. (2002). The proliferative ventricular zone in adult vertebrates: a comparative study using reptiles, birds, and mammals. *Brain Res. Bull.* 57 765–775. 10.1016/S0361-9230(01)00769-9 12031273

[B26] González-Granero S. Alfaro-Cervelló C. Capilla-González V. Lezameta-Morgan M. Romaguera-Ros M. García-Verdugo J. M. (2008). “Editorial: Neurogenic sites in non-mammalian vertebrates,” in *Postnatal and Adult Neurogensis*, (Thiruvananthapuram: Research Signpost), 249–273.

[B27] Holtzman D. A. Halpern M. (1991). Incorporation of 3H-thymidine in telencephalic structures of the vomeronasal and olfactory systems of embryonic garter snakes. *J. Comp. Neurol.* 304 450–466. 10.1002/cne.903040309 2022759

[B28] Jacyniak K. McDonald R. P. Vickaryous M. K. (2017). Tail regeneration and other phenomena of wound healing and tissue restoration in lizards. *J. Exp. Biol.* 220 2858–2869. 10.1242/jeb.126862 28814609

[B29] Kaplan M. S. Hinds J. W. (1977). Neurogenesis in the adult rat: electron microscopic analysis of light radioautographs. *Science* 197 1092–1094. 10.1126/science.887941 887941

[B30] Lazzari M. Franceschini V. (2001). Glial fibrillary acidic protein and vimentin immunoreactivity of astroglial cells in the central nervous system of adult *Podarcis sicula* (Squamata. Lacertidae). *J. Anat.* 198 67–75. 10.1046/j.1469-7580.2001.19810067.x 11215769 PMC1468192

[B31] Lois C. Alvarez-Buylla A. (1994). Long-distance neuronal migration in the adult mammalian brain. *Science* 264 1145–1148. 10.1126/science.8178174 8178174

[B32] Lois C. Garcia-Verdugo J. M. Alvarez-Buylla A. (1996). Chain migration of neuronal precursors. *Science* 271 978–981. 10.1126/science.271.5251.978 8584933

[B33] Lopez-Garcia C. Molowny A. Garcia-Verdugo J. M. Ferrer I. (1988). Delayed postnatal neurogenesis in the cerebral cortex of lizards. *Brain Res.* 471 167–174. 10.1016/0165-3806(88)90096-X 3179748

[B34] Lopez-Garcia C. Molowny A. Garcia-Verdugo J. M. Martinez-Guijarro F. J. Bernabeu A. (1990). Late generated neurons in the medial cortex of adult lizards send axons that reach the timm-reactive zones. *Brain Res. Dev. Brain Res.* 57 249–254. 10.1016/0165-3806(90)90050-9 2073723

[B35] López-García C. Tineo P. L. Del Corral J. (1984). Increase of the neuron number in some cerebral cortical areas of a lizard, *Podarcis hispanica*, (Steind., 1870), during postnatal periods of life. *J. Hirnforsch.* 25 255–259. 6470463

[B36] Luis de la Iglesia J. A. Lopez-Garcia C. (1997). A Golgi study of the principal projection neurons of the medial cortex of the lizard *Podarcis hispanica*. *J. Comp. Neurol.* 385 528–564. 10.1002/(SICI)1096-9861(19970908)385:4<528::AID-CNE4>3.0.CO;2-5 9302105

[B37] Marchioro M. Nunes J.-M. de A. M. Ramalho A. M. R. Molowny A. Perez-Martinez E. (2005). Postnatal neurogenesis in the medial cortex of the tropical lizard *Tropidurus hispidus*. *Neuroscience* 134 407–413. 10.1016/j.neuroscience.2005.04.014 15961247

[B38] McDonald R. P. Vickaryous M. K. (2018). Evidence for neurogenesis in the medial cortex of the leopard gecko. *Eublepharis macularius*. *Sci. Rep.* 8:9648. 10.1038/s41598-018-27880-6 29941970 PMC6018638

[B39] Medina L. Abellán A. Desfilis E. (2017). Contribution of genoarchitecture to understanding hippocampal evolution and development. *Brain Behav. Evol.* 90 25–40. 10.1159/000477558 28866679

[B40] Merkle F. T. Tramontin A. D. Garcia-Verdugo J. M. Álvarez-Buylla A. (2004). Radial glia give rise to adult neural stem cells in the subventricular zone. *Proc. Natl. Acad. Sci. U S A.* 101 17528–17532. 10.1073/pnas.0407893101 15574494 PMC536036

[B41] Nottebohm F. Alvarez-Buylla A. (1993). Neurogenesis and neuronal replacement in adult birds. *Neuronal Cell Death Repair* 31 227–236. 10.1016/B978-0-444-81470-8.50022-3

[B42] Olucha F. Martinez-Garcia F. Poch L. Schwerdtfeger W. K. Lopez-Garcia C. (1988). Projections from the medial cortex in the brain of lizards: correlation of anterograde and retrograde transport of horseradish peroxidase with Timm staining. *J. Comp. Neurol.* 276 469–480. 10.1002/cne.902760402 2461968

[B43] Paredes M. F. Sorrells S. F. García-Verdugo J. M. Álvarez-Buylla A. (2016). Brain size and limits to adult neurogenesis. *J. Comp. Neurol.* 524 646–664. 10.1002/cne.23896 26417888 PMC5047485

[B44] Pastrana E. Cheng L.-C. C. Doetsch F. (2009). Simultaneous prospective purification of adult subventricular zone neural stem cells and their progeny. *Proc. Natl. Acad. Sci. U S A.* 106 6387–6392. 10.1073/pnas.0810407106 19332781 PMC2669396

[B45] Peñafiel A. Gutiérrez A. Martín R. Mar Pérez-Cañellas M. de la Calle A. (1996). A tangential neuronal migration in the olfactory bulbs of adult lizards. *Neuroreport* 7 1257–1260. 10.1097/00001756-199605170-00007 8817544

[B46] Pérez-Canellas M. M. García-Verdugo J. M. (1992). Adult neurogenesis in reptiles: a comparative study using [3H]thymidine autoradiography. *Eur. J. Neurosci.* S5:294.

[B47] Pérez-Cañellas M. M. Font E. Garcia-Verdugo J. M. (1997). Postnatal neurogenesis in the telencephalon of turtles: evidence for nonradial migration of new neurons from distant proliferative ventricular zones to the olfactory bulbs. *Brain Res. Dev. Brain Res.* 101 125–137. 10.1016/S0165-3806(97)00058-8 9263587

[B48] Pérez-Cañellas M. M. García-Verdugo J. M. (1996). Adult neurogenesis in the telencephalon of a lizard: a [^3^H]thymidine autoradiographic and bromodeoxyuridine immunocytochemical study. *Brain Res. Dev. Brain Res.* 93 49–61. 10.1016/0165-3806(96)00014-4 8804691

[B49] Pimentel H. de C. Macêdo-Lima M. Viola G. G. Melleu F. F. Dos Santos T. S. (2021). Telencephalic distributions of doublecortin and glial fibrillary acidic protein suggest novel migratory pathways in adult lizards. *J. Chem. Neuroanat.* 112:101901. 10.1016/j.jchemneu.2020.101901 33271217

[B50] Polenov A. L. Chetverukhin V. K. (1993). Ultrastructural radioautographic analysis of neurogenesis in the hypothalamus of the adult frog, Rana temporaria, with special reference to physiological regeneration of the preoptic nucleus. *Cell Tissue Res.* 271 351–362. 10.1007/BF00318622 8453659

[B51] Quinn G. P. Keough M. J. (2002). *Experimental Design and Data Analysis for Biologists.* Cambridge: Cambridge University Press. 10.1017/CBO9780511806384

[B52] Sampedro C. Font E. Desfilis E. (2008). Size variation and cell proliferation in chemosensory brain areas of a lizard (*Podarcis hispanica*): effects of sex and season. *Eur. J. Neurosci.* 28 87–98. 10.1111/j.1460-9568.2008.06287.x 18662337

[B53] Sanai N. Tramontin A. D. Quiñones-Hinojosa A. Barbaro N. M. Gupta N. Kunwar S. (2004). Unique astrocyte ribbon in adult human brain contains neural stem cells but lacks chain migration. *Nature* 427 740–744. 10.1038/nature02301 14973487

[B54] Schulz E. (1969). Postnatal biomorphosis of the ependyma in the telencephalon of *Lacerta agilis agilis*. *Z. Mikrosk. Anat. Forsch.* 81 111–152. 4906482

[B55] Seri B. García-Verdugo J. M. McEwen B. S. Alvarez-Buylla A. (2001). Astrocytes give rise to new neurons in the adult mammalian hippocampus. *J. Neurosci.* 21 7153–7160. 10.1523/JNEUROSCI.21-18-07153.2001 11549726 PMC6762987

[B56] Shao H. Fan L. Xu X. J. Xu W. Q. Liu B. F. Wang J. L. (2012). Characterization of adult neurogenesis in lizard *Phrynocephalus vlangalii* (Agamidae: Reptilia). *Ital. J. Zool.* 79 547–558. 10.1080/11250003.2012.719933

[B57] Tineo P. L. Planelles M. D. Del-Corral J. (1987). Modifications in cortical ependyma of the lizard, *Podarcis hispanica*, during postnatal development. *J. Hirnforsch.* 28 485–489. 3693891

[B58] Tramontin A. D. García-Verdugo J. M. Lim D. A. Alvarez-Buylla A. (2003). Postnatal development of radial glia and the ventricular zone (VZ): a continuum of the neural stem cell compartment. *Cereb. Cortex* 13 580–587. 10.1093/cercor/13.6.580 12764031

[B59] yanes-méndez c. martin-trujillo j. m. pérez-batista m. a. monzón-mayor m. marrero a. (1988a). ependymogenesis of the lizard basal Areas 2. sulcus. *Z. Mikrosk. Anat. Forsch.* 102 573–589.

[B60] Yanes-Méndez C. Martin-Trujillo J. M. Pérez-Batista M. A. Monzón-Mayor M. Marrero A. (1988b). Ependymogenesis of the lizard basal Areas 1. ependymal zones. *Z. Mikrosk. Anat. Forsch.* 102 555–572.

[B61] Zupanc G. K. (2001). A comparative approach towards the understanding of adult neurogenesis. *Brain Behav. Evol.* 58 246–249. 10.1159/000057568 11978944

[B62] Zupanc G. K. Horschke I. (1995). Proliferation zones in the brain of adult gymnotiform fish: a quantitative mapping study. *J. Comp. Neurol.* 353 213–233. 7745132 10.1002/cne.903530205

